# Computational design of highly stable and soluble alcohol dehydrogenase for NADPH regeneration

**DOI:** 10.1186/s40643-021-00362-w

**Published:** 2021-02-07

**Authors:** Jinling Xu, Haisheng Zhou, Haoran Yu, Tong Deng, Ziyuan Wang, Hongyu Zhang, Jianping Wu, Lirong Yang

**Affiliations:** 1 Institute of Bioengineering, College of Chemical and Biological Engineering, Zhejiang University, Hangzhou, 310027 China; 2 Hangzhou Global Scientific and Technological Innovation Center, Zhejiang University, Hangzhou, 310027 China

**Keywords:** Alcohol dehydrogenase, Computational design, Soluble expression, NADPH regeneration, Chiral alcohols

## Abstract

Nicotinamide adenine dinucleotide phosphate (NADPH), as a well-known cofactor, is widely used in the most of enzymatic redox reactions, playing an important role in industrial catalysis. However, the absence of a comparable method for efficient NADP^+^ to NADPH cofactor regeneration radically impairs efficient green chemical synthesis. Alcohol dehydrogenase (ADH) enzymes, allowing the in situ regeneration of the redox cofactor NADPH with high specific activity and easy by-product separation process, are provided with great industrial application potential and research attention. Accordingly, herein a NADP^+^-specific ADH from *Clostridium beijerinckii* was selected to be engineered for cofactor recycle, using an automated algorithm named Protein Repair One-stop Shop (PROSS). The mutant CbADH-6M (S24P/G182A/G196A/H222D/S250E/S254R) exhibited a favorable soluble and highly active expression with an activity of 46.3 U/mL, which was 16 times higher than the wild type (2.9 U/mL), and a more stable protein conformation with an enhanced thermal stability: Δ ${T}_{1/2}^{60\mathrm{min}}$=  + 3.6 °C (temperature of 50% inactivation after incubation for 60 min). Furthermore, the activity of CbADH-6M was up-graded to 2401.8 U/mL by high cell density fermentation strategy using recombinant *Escherichia coli*, demonstrating its industrial potential. Finally, the superb efficiency for NADPH regeneration of the mutant enzyme was testified in the synthesis of some fine chiral aromatic alcohols coupling with another ADH from *Lactobacillus kefir* (LkADH).

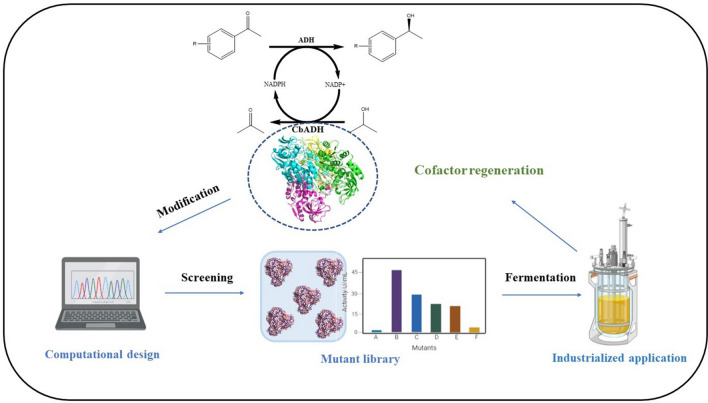

## Introduction

**Fig. 1:**
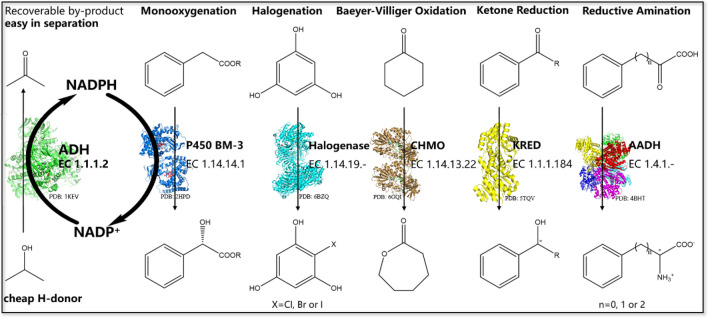
Biosynthetic reactions using NADPH

The ability of enzymes to operate simply in aqueous systems in a highly efficient manner makes them attractive environmentally benign synthetic reagents. However, many biocatalysts have not been fully exploited in industry, and their use in the large-scale enzymatic synthesis of high added-value chemicals is often limited by expensive cofactors. Typical cofactor dependent enzymes are oxidoreductases representing around 25% of all known enzymes (Liu and Wang 2007), which catalyzes about 30% of the biotransformations in industry (Straathof et al. 2002), and the vast majority are dependent on two nicotinamide cofactors NADH or NADPH. Although these two cofactors differ only by the 2′-phosphate group that is attached to the adenine ribose in NADPH, they play a completely different role in nature. NADH is used almost exclusively for oxidative degradations that eventually lead to production of ATP, whereas NADPH is confined with few exceptions to the biosynthetic reactions (Carugo and Argos 1997), involving a spectrum of over 300 known, repeatedly used reaction types (Woodyer et al. 2005), e.g., C–H oxygenation (Landwehr et al. 2006), regioselective halogenation (Mori et al. 2019), Baeyer–Villiger oxidation (Schmidt et al. 2015), stereoselective reduction (Zhu and Hua 2006) and reductive amination (Yin et al. 2020) (Fig. 1). Therefore, it is necessary to develop an efficiently applicable technique for the in situ regeneration of NADPH to fulfill “green” chemical synthesis.

Currently, numerous chemical, electrochemical, photochemical and biochemical methods have been developed to regenerate NADPH (Brown et al. 2016; Fukuzumi et al. 2019; Wichmann and Vasic-Racki 2005). Most chemical routes are hindered by cumbersome reaction conditions, low turnover number (TTN), expensive and/or toxic reagents, and/or unwanted side products, therefore not preferred for commercial or preparative applications. Biochemical methods with enzymes as catalysts have been demonstrated more efficient and applicable (Huisman et al. 2010), including the use of glucose dehydrogenase (GDH) (Weckbecker and Hummel 2005), glucose-6-phosphate dehydrogenase (G6PDH) (Zhang et al. 2006), alcohol dehydrogenase (ADH) (Bastos et al. 1999), phosphite dehydrogenase (PDH) (Johannes et al. 2007a), formate dehydrogenase (FDH) (Celik et al. 2014), hydrogenase (Fan et al. 2020) and so forth. GDH and G6PDH with an advantage of high specific activity (200–500 U/mg at 25–30 °C) (Ding et al. 2011; Wennekes et al. 1993) suffer from high co-substrate consumption and by-product separation cost. By contrast, PDH and FDH using cheap co-substrate to regenerate NADPH without by-product problem, possess very low specific activity (FDH, 5–20 U/mg at 25–30 °C; PDH, 5 U/mg at 25 °C) (Vázquez-Figueroa et al. 2007). Additionally, because of significantly low stability and activity, the biotechnological applications of hydrogenases are still in their infancy (Fan et al. 2020). Nevertheless, ADH with a comparable specific activity (140–170 U/mg at 25–40 °C) (Peretz et al. 1997; Widdel and Wolfe 1989) and easy by-product separation process when using isopropanol as cheap H-donor (Fig. 1), is of considerable commercial value as a catalyst for NADPH regeneration in the synthesis and/or biotransformation of valuable compounds.

However, NADPH-dependent ADHs have been widely applied to the synthesis of chiral alcohols and few studies applied the ADH for NADPH coenzyme recycle (Benítez-Mateos et al. 2017; He et al. 2015; Itoh 2014). Although some ADHs can catalyze the synthesis of product and the regeneration of cofactor simultaneously in a substrate-coupled system, the two substrates could be competitively inhibited by each other, which decreased the production of the target products. Moreover, advanced enzyme engineering technologies have been used to improve some ADH performances including substrate specificity, enantioselectivity and catalytic activity. However, the NADPH regenerating ADHs have not been explored.

Here an ADH from *Clostridium beijerinckii* (CbADH) was identified with excellent thermostability and high specific activity for NADPH regeneration. In order to improve the poor heterologous expression level of CbADH (Peretz et al. 1997), an automated structure- and sequence-based computational protein redesign method named Protein Repair One-Stop Shop (PROSS) (Goldenzweig et al. 2016), was applied to engineer wild-type CbADH to obtain improved mutants that could satisfy laboratory and even industrial applications. Furthermore, a two-phase high cell density fermentation strategy was explored to the large-scale production of the best mutant to demonstrate its industrial potential. Also, the performance of the obtained variant was verified at in situ NADPH regeneration in the synthesis of some fine chiral aromatic alcohols coupling with another engineered ADH from *Lactobacillus kefir* (LkADH) (He et al. 2015).

## Materials and methods

### Microorganisms and plasmids

An in-house *E. coli* BL21 (DE3) was used as a host bacteria for recombinant expression The plasmid pET-28a (with N-terminal His-tag fused) carrying the TgADH, CbADH, TbADH, TeADH, EhADH and LkADH gene were prepared by Beijing Tsingke Biological Technology Company, who also carried out the primer synthesis and sequencing.

### Culture and induction conditions

The recombinant *E. coli* cells were first cultured for 6–8 h at 37 ℃ in 5 mL Luria–Bertani (LB) medium supplemented with 50 μg/mL kanamycin sulfate, which were then transformed into flask (50 mL LB) at 37℃ with rotary shaking at 200 rpm until the OD_600_ reached 0.4–0.6. Cells were induced at the specific temperature (16–30 ℃)for 16 h by addition of isopropyl-β-D-thiogalactopyranoside (IPTG, 0.1–1.0 mM).

### Construction of PROSS mutants

The CbADH-related parameters and protein crystal structure were submitted online on a dedicated web server (http://PROSS.weizmann.ac.il). The recombinant plasmid pET-28a-CbADH containing the CbADH mutants were constructed through whole gene synthesis, and expressed in *E. coli* BL21. All sequences were verified by DNA sequencing at Beijing Tsingke biological technology Co. Ltd.

### Enzyme assay

The induced cells were harvested by centrifugation and washed three times using deionized water. Finally, the harvested cells were resuspended in 100 mM phosphate buffer (pH 7.5) and disrupted ultrasonically. The obtained suspension was diluted for enzyme assay. The standard assay mixture (1 mL) consisted of 100 mM phosphate buffer (pH 7.5), 50 mM isopropyl alcohol, 1 mM NADP^+^, and enzyme. The substrate and coenzyme solution were incubated in a metal bath at 35 ℃ and 650 rpm for 10 min. Once the enzyme solution was added, the reaction solution was scanned at 340 nm by spectrophotometer for 60 s, and the change in absorbance was recorded to calculate the enzyme activity. One unit of enzyme activity was defined as the amount of enzyme required to catalyze the formation of 1 μmol NADPH per minute.

### SDS-PAGE and protein concentration assays

The expression and purification of the recombinant ADHs were analyzed by sodium dodecyl sulfate–polyacrylamide gel electrophoresis (SDS-PAGE, 12%) with a 5% stacking gel. The gels were stained with Coomassie Brilliant Blue G-250. Protein concentrations were determined using a Bradford protein assay kit (Quick Start™, Bio-Rad, USA).

### Purification of recombinant protein

The collected cells were washed and resuspended in Ni-0-native buffer (20 mM sodium phosphate, pH 7.5, containing 500 mM NaCl). Resuspended cells were disrupted by ultrasonication in an ice bath, followed by centrifugation at 12,000*g* for 30 min to discard cell debris. The supernatant was loaded onto a Ni–NTA column (Thermo Scientific, USA) pre-equilibrated with Ni-0-native buffer, and the proteins were eluted by an increasing gradient of imidazole (from 50 to 250 mM). The purities of the collected fractions were analyzed by SDS-PAGE. Fractions containing the pure target protein were gathered and desalted by ultrafiltration. The purified proteins were concentrated and stored in 20% (v/v) glycerol at − 80 °C until further use.

### Characterization of enzymatic properties

Temperature and pH dependence: the temperature dependence was determined over the range 15–75 °C. The mixture except the enzyme was preincubated for 10 min at a serious of temperature, and the reaction was initiated by the addition of enzyme solution preincubated for 10 min at the same temperature. The optimum pH was determined at 35 °C using different buffer systems to cover the pH scale: 100 mM phosphate buffer (pH 6.0–7.5); 100 mM Tris–HCl buffer (pH 7.5–9.0); 100 mM glycine–NaOH buffer (pH 9.5–11.0); 100 Mm Na_2_HPO_4_ buffer (pH 11.0–13.0).

pH stability: the pH stability was measured by incubation of purified enzyme in buffer (pH 6–10) for 24 h at 35 °C, and the residual activity was then measured by standard assay.

Thermal stability: the thermal stability was characterized by ${T}_{1/2}^{60\mathrm{min}}$ (temperature of 50% inactivation after incubation for 60 min) and half-life. ${T}_{1/2}^{60\mathrm{min}}$ was measured at different temperature where the enzyme activity is reduced to 50% of its initial activity after incubation for 60 min. The half-life (*t*_1/2_) was defined as the time when the residual activity retained 50% of its original activity at the measured temperature. The diluted purified CbADH solution (1.0 μg/mL) was preincubated at 50, 60, 70 ℃ for different durations. The residual enzyme activity was then measured under standard conditions.

### High-density fermentation

Single colonies were selected from the plate and incubated in 50 mL LB medium for primary seeds, and cultured overnight at 37 ℃, 200 rpm. The cells were then transferred to 500 mL LB medium for secondary seeds and cultured at 200 rpm, 37 ℃ for 4 h. The secondary seed culture solution was transferred to a 15.0-L fermentation tank under aseptic conditions, cultured in a 7.0-L fermentation medium (15.0 g/L glycerol; 25.0 g/L yeast extract (Xiwang, China); 17.1 g/L Na_2_HPO_4_·12H_2_O; 3.0 g/L KH_2_PO4; 1.0 g/L NaCl; 1.0 g/L NH_4_Cl; 1.3 g/L MgSO_4_·7H_2_O; 0.3 g/L ZnSO_4_·7H_2_O; and 0.05 g/L kanamycin sulfate;) at 37 ℃. When the glycerol of the fermentation medium was depleted, the feeding medium (400.0 g/L glycerol; 100.0 g/L yeast extract) was started. To avoid the accumulation of glycerol in the broth, the feeding medium was added using a continuous model by monitoring the changes in the DO (dissolved oxygen) level, pH value and glycerol concentration during fermentation. The tank was cooled to 22 ℃ for induction until the cell density (OD_600_) reached 70–90. The pH was controlled at 7.0 through the adjustment of ammonia, additionally the dissolved oxygen was controlled at about 30%.

### HPLC analysis and detection

The conversion of ketones and the enantiomeric excess of chiral alcohol products were detected with a normal-phase chiral column (CHIRAL PAK, ZWIX(−), 3 μm, 4 × 150 mm) using a FL-2200 HPLC system (Fuli Analytical Instrument Co., Ltd., China). The flow rate was maintained at 0.6 mL/min, with detection at 230 nm, and the column temperature was constant at 25 ℃, applying isocratic elution of n-hexane/isopropanol/trichloroacetic acid (90/10/0.1, v/v/v).

### Preparative applications of ADHs for the synthesis of chiral alcohols

Reactions were carried out at 35 °C by magnetic agitation with 100 mM phosphate buffer (pH 7.5) as the medium (50 mL). Each reaction sample contained 50 mM precursor ketone, 60 mM isopropanol, 0.1 mM NADP^+^, 1.0 gdcw L^−1^ LkADH cells for LkADH mono-enzymatic reaction or 0.95 gdcw L^−1^ LkADH cells plus 0.05 gdcw L^−1^ CbADH-6M cells for dual enzyme catalyzed reaction. The recombinant LkADH and CbADH-6M cells were resuspended in 100 mM phosphate buffer (pH 7.5) and disrupted ultrasonically. The resultant slurry was centrifuged at 12,000*g* and the supernatant was used for the redox reaction. The reaction process was monitored by the determination of residual substrate concentration using HPLC, as described in the “HPLC analysis and detection” section.

## Results and discussion

### Alcohol dehydrogenase library construction and activity identification

**Fig. 2:**
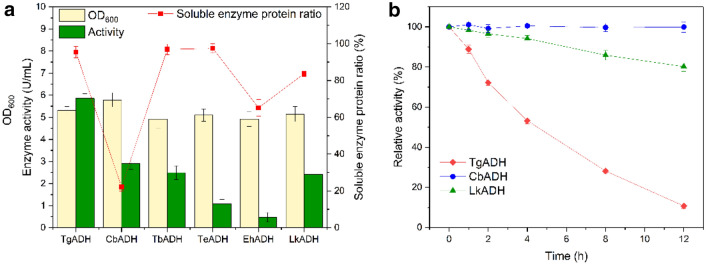
Characterization of alcohol dehydrogenases. **a** Enzyme activity and soluble expression of alcohol dehydrogenases in shake-flask fermentation at 25 ℃ with 0.5 mM IPTG dosage. **b** Stability of the crude extract enzymes in atmosphere at 25 °C

NADPH-specific ADH (EC 1.1.1.2) from different sources have been reported a lot according to the enzyme database BRENDA. However, few of them have high activity. Here, an ADH library containing CbADH with specific activity exceeding about 10 U/mg was constructed (Additional file 1: Table S1). However, some of them were not suitable for NADPH regeneration as they only oxidized primary alcohols and produced aldehydes which inactivated enzymes. Hence, those showing activity towards secondary alcohols were selected for further investigation. Soluble expression levels and NADPH regenerating activities (per volume of fermentation broth) were tested for these secondary ADHs with an exception of MtADH (Additional file 1: Fig. S1), which uses unavailable F_420_ as a cofactor (Widdel and Wolfe 1989). All the other six enzymes were successfully expressed in *E. coli* (Additional file 1: Fig. S2) and considerable activities of TgADH, CbADH, TbADH and LkADH were obtained (Fig. 2a). Among them, TgADH exhibited a highest activity but a limited stability in atmosphere, meanwhile LkADH was also demonstrated to be less stable than CbADH (Fig. 2b). CbADH showed the second highest activity (2.9 U/mL) with a soluble enzyme protein ratio of 25.1%, which was the lowest and less than a quarter of TbADH (96.9%), indicating that the specific activity of CbADH was supreme. Subsequently, the recombinant CbADH was purified to electrophoretic purity with an activity of 169 ± 2.3 U/mg. Considering its high specific activity and stability, CbADH was selected for NADPH regeneration, despite that the heterogenous expression of CbADH was urgently needed to be improved.

### Culturing conditions optimization for CbADH expression

**Fig. 3:**
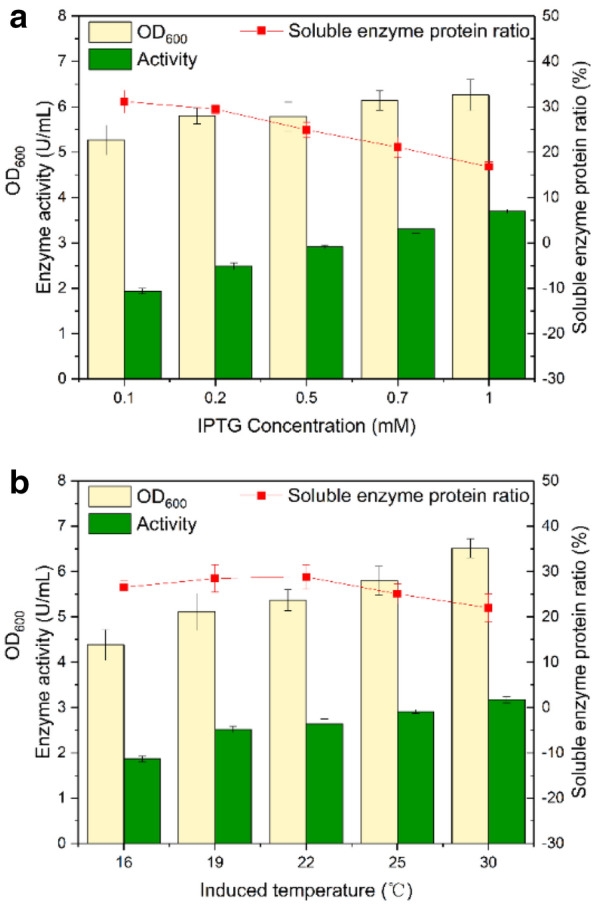
Effects of induction conditions on the wild CbADH activity in shake-flask fermentation. **a** Induced by different IPTG concentrations (0.1–1.0 mM) at 25 ℃. **b** Induced at different temperatures (16–30 ℃) with 0.5 mM IPTG dosage

The most conventional and simple method to improve the heterogenous protein expression is to balance the target protein biosynthesis and the cell growth by optimizing the induction temperature and the concentration of the inducer IPTG (Donovan et al. 1996). However, the effect of this culture condition optimization strategy on the expression of CbADH was not significant. With the increase of IPTG concentration, the total enzyme activity increased slightly, but the proportion of soluble enzyme protein decreased (Fig. 3a and Additional file 1: Fig. S4). Additionally, the induction temperature had little influence on the activity and soluble enzyme–protein ratio (Fig. 3b and Additional file 1: Fig. S3). The maximum enzyme activity was only 3.7 U/mL with the lowest soluble target protein ratio of 16.8%, achieved at induction temperature 25 °C and IPTG concentration of 1 mM.

### Improving CbADH solubility by chaperone buffering

Chaperone-assisted protein folding was another frequently used approach to overcome the problem of low soluble expression in overexpression systems (Balbás 2001). Therefore, five commercial chaperones were co-expressed with CbADH, respectively, and the results showed that strain A co-expressed with GroEL/ES preferably improved the soluble expression of CbADH with an activity of 14.1 U/mL and a soluble target protein ratio of about 59.6% (Additional file 1: Fig. S5, 6). Subsequently, the chaperone co-expression was optimized and the results were shown in another of our work (Deng et al. 2020). However, the expression of chaperone protein will occupy the resources of cells for expressing target enzyme protein, as a result, the target enzyme activity could not reach the theoretical maximum.

### Computational protein redesign for enhancing CbADH solubility

**Fig. 4:**
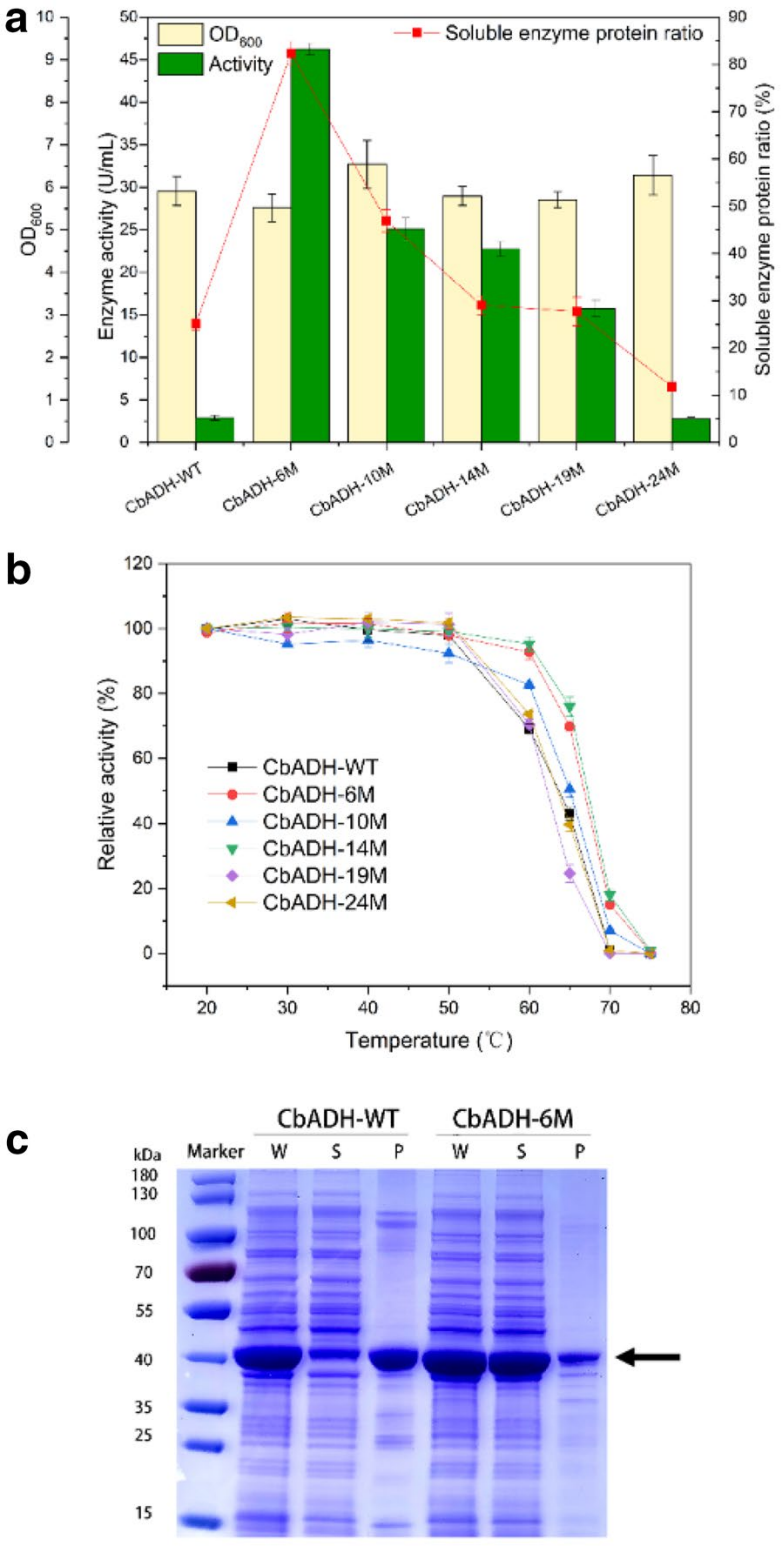
Characterization of CbADH mutants compared with the wild type. **a** Enzyme activity in shake-flask fermentation at 25 ℃ with 0.5 mM IPTG dosage. The green column represents the crude enzyme activity, while the beige column represents OD_600_. The red square represents the proportion of soluble protein to total protein. **b** Thermal stability of the wild CbADH and the mutants. **c** SDS-PAGE analysis of protein expression of the wild-type CbADH-WT and mutant CbADH-6M: Lane M: molecular weight marker; Lane W: whole cell protein; Lane S: supernatant; Lane P: precipitation

The gap between the high specific activity versus the extremely low expressional activity becomes a major obstacle to the industrial application of CbADH. Directed evolution and computational redesign of natural enzymes have proven capable of bridging equally large gaps (Musil et al. 2018). Previous studies have used site-directed mutagenesis to improve the stability of CbADH (Bogin et al. 2002; Goihberg et al. 2007), but neglected its soluble expression. Unfortunately, stability enhancements often come at the cost of reduced enzyme activity. The computational protein redesign tools provided another method to avoid this trade-off and also to solubilize the polypeptides, facilitating the purposeful adaptation of natural enzymes. Among them, Protein Repair One-Stop Shop (PROSS), an automated web-based protein stabilization platform was proven efficient by the soluble expression of several human proteins in bacteria with unmodified function (Goldenzweig et al. 2016). Here, the PROSS was applied to redesign CbADH. Functionally relevant key sites, including Zn^2+^ and cofactor ligand binding sites, and dimer interface sites were excluded from mutation (Additional file 1: Fig. S7, Table S3). After three rounds of online calculations, five random mutations were selected for experimental verification. Synthetic genes encoding wild-type CbADH (CbADH-WT) and the five designs were expressed in *E. coli* BL21 (DE3), and the results showed that mutations except CbADH-24M were expressed with higher solubility and activity than the wild-type CbADH, and the soluble enzyme protein ratio and activity dramatically decreased with the number of mutation sites increased (Fig. 4a and Additional file 1: Fig. S8), that was different from Goldenzweig’s work. In addition, thermal stabilities of three mutations, CbADH-6M (${T}_{1/2}^{60\mathrm{min}}$=66.8 °C), CbADH-10M (${T}_{1/2}^{60\mathrm{min}}$=64.9 °C) and CbADH-14M (${T}_{1/2}^{60\mathrm{min}}$=67.3 °C) were significantly improved compared with the wild-type enzyme (${T}_{1/2}^{60\mathrm{min}}$=63.7 °C) (Fig. 4b). The best variant CbADH-6M was tested to have an expressional activity of 46.3 U/mL and a soluble enzyme protein ratio of 82.4%, which were 16-fold and threefold higher than the wild type, respectively (Fig. 4c). Although completely soluble expression was not achieved by sequence redesign of CbADH, PROSS has been proved to be a simple and applicable method for computational design of stable and soluble biocatalysts.

### Molecular structure analysis of CbADH-6M

**Fig. 5:**
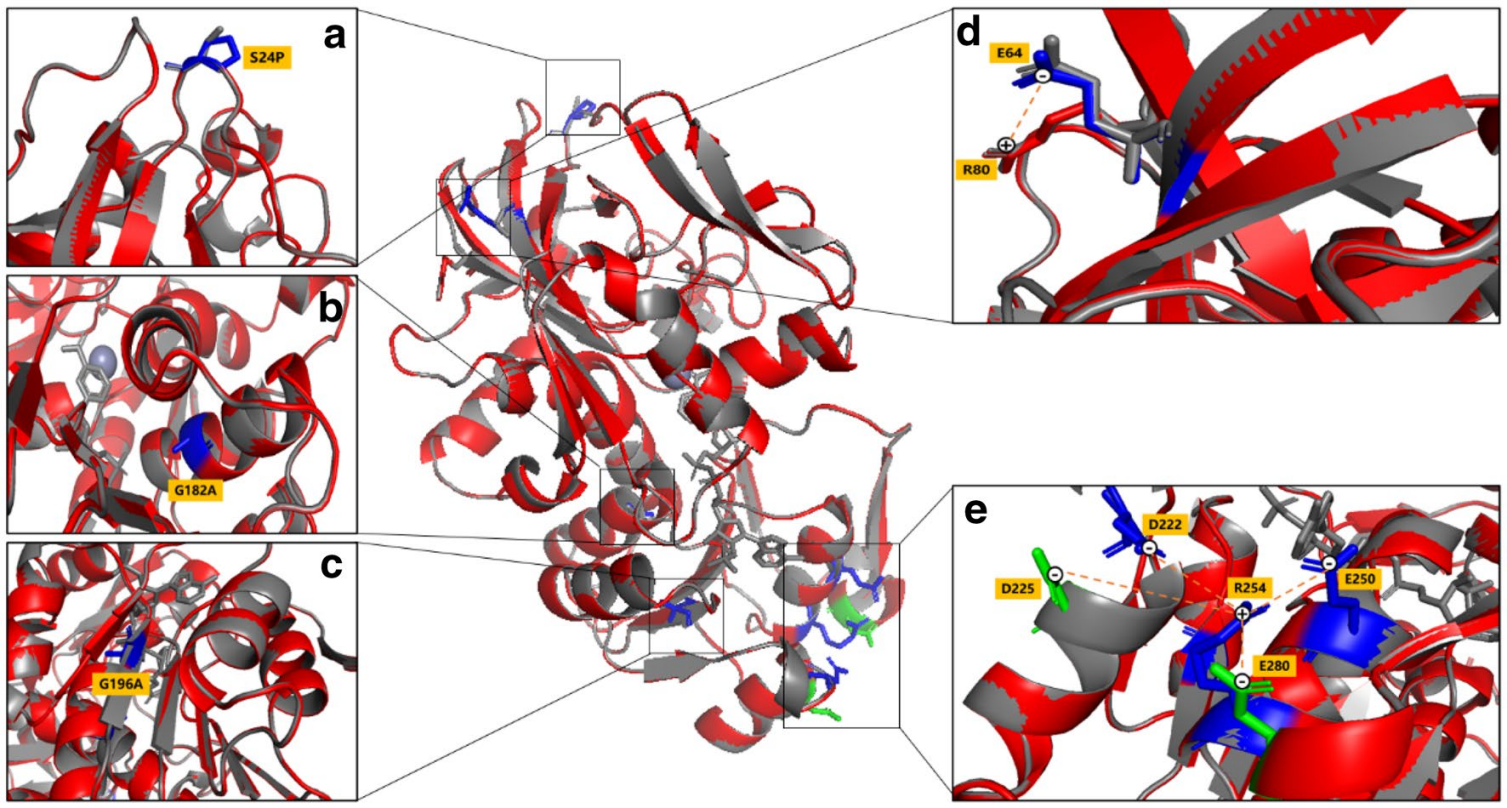
Protein structure of CbADH-6M (red backbone) compared with the wild CbADH (gray backbone). **a** The mutant S24P located in the loop region. **b** The mutant G182A located in the α-helix. **c** The mutant G196A located at β-sheets. **d** New salt bridge Glu64–Arg80 formed by CbADH-6M. **e** Schematic diagram of new salt bridge network centered on Arg254

Multiple superimposable mutations can improve the conformational stability of the protein in its natural state, allowing it to gain an advantage in the competition with other misfolded or partially folded states in folding dynamics. Six mutations, S24P, G182A, G196A, H222D, S250E and S254R were introduced into the CbADH-6M mutant. Among the 6 mutation sites involved in CbADH-6M, four sites including 24, 222, 250 and 254 were located on the surface of the protein (Fig. 5). Three mutations including H222D, S250E, and S254R replaced the original amino acid residues with more polar amino acid residues which enhanced the surface polarity. In addition, the significant increase of ${T}_{1/2}^{60\mathrm{min}}$ gained by the substitution of Pro for Gln100 in CbADH (△${T}_{1/2}^{60\mathrm{min}}$ =  + 8 ℃) (Musil et al. 2018) suggested that the proline residue stabilized the protein by reducing the flexibility of a loop at this strategic region. Similarly, when S24 located in a surface loop of the protein was mutated to a proline residue that adopted only a limited number of conformations, the flexibility of the loop was reduced thus rigidifying the protein structure. As to the G182 located in the internal structure of an alpha helix, it was substituted by alanine residue (López-Llano et al. 2006), which is regarded as the most stabilizing residue in internal helical position, whereas glycine is the more destabilizing after proline. Furthermore, it was found that the number of salt bridges in CbADH-6M changed a lot. Although the Asp225–His222 salt bridge in the original protein CbADH-WT disappeared due to the amino acid substitution H222D, the replacement of H222D, S250E and S254R led to the formation of four new salt bridges including Arg254–Glu250, Arg254–Asp225, Arg254–Glu280 and Arg254–Asp222, which constituted a salt bridge network centered on Arg254. Moreover, the angle of the Arg80 changed, making it closer to the Glu60 to form a new salt bridge between Glu64 and Arg80.

### Large-scale production of CbADH-6M

**Fig. 6:**
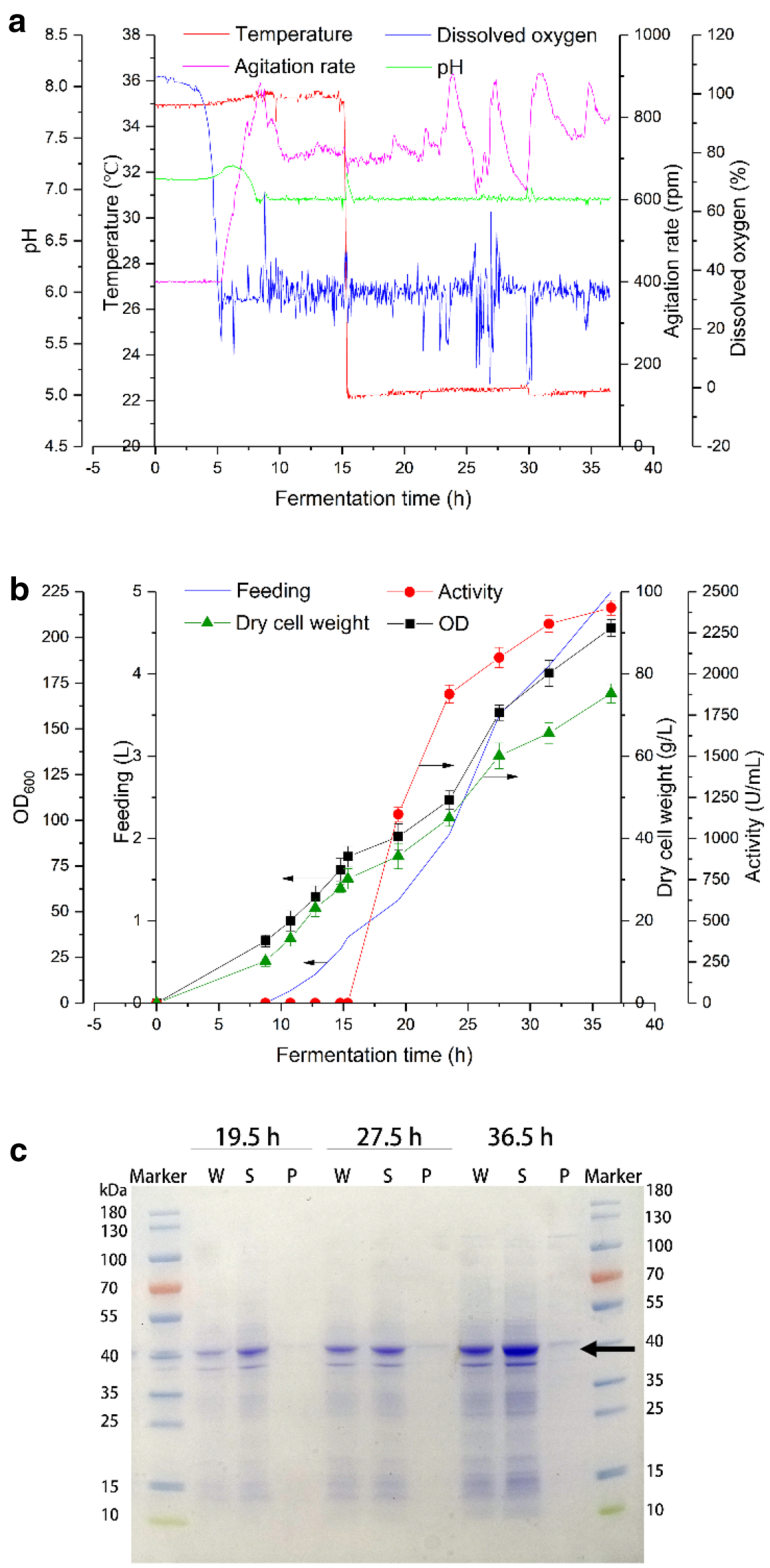
Fermentation process of recombinant *Escherichia coli*-pET-28a-CbADH-6M in a 15.0-L mechanically stirred fermentor. **a** Fermentation parameter control. **b** Cell growth and CbADH-6M expression during the fermentation. **c** SDS-PAGE analysis of protein expression of samples at different time: Lane M: molecular weight marker; Lane W: whole cell protein; Lane S: supernatant; Lane P: precipitation

In a two-phase high cell density fermentation, the dissolved oxygen dropped sharply in 5 h, and the agitation rate increased at the same time. The other fermentation parameters are shown in Fig. 6a. Sampling was started at 9 h, and the measured OD_600_ reached 80.1 at 15 h, and the induction temperature was 22 ℃. During the subsequent fed fermentation, glycerol was used as carbon source instead of glucose, the utilization of which by *E. coli* cells was faster and more complete. Moreover, the accumulation of acetic acid in the process was effectively controlled near zero by the feeding strategy of dissolved oxygen feedback, so the main inhibiting factor of the growth of *E. coli* cells was eliminated and the enzyme activity and OD_600_ could increase steadily (Fig. 6b). At the end of the fermentation (36.5 h), the OD_600_ reached 205.2 and the enzyme activity reached 2401.8 U/mL (31.9 U/mg dcw). As shown in the SDS-PAGE, the sampling was carried out at 19.5 h, 27.5 h and 36.5 h and the amount of total protein and soluble protein continued to increase as the fermentation proceeded. And almost no precipitation was observed indicating the excellent soluble expression achieved (Fig. 6c).

### Biochemical characterization of recombinant CbADH-6M

**Fig. 7:**
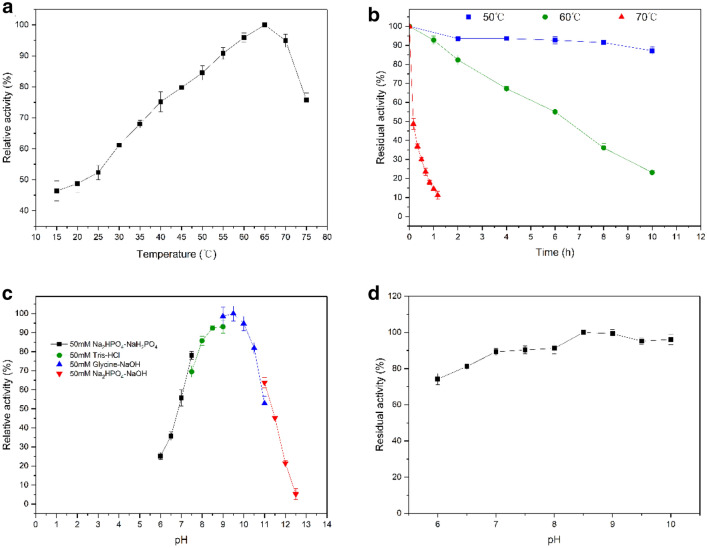
Characterization of the recombinant CbADH-6M. **a** Temperature optimum: the enzyme activity was measured at various temperatures (15–75 ℃) in phosphate buffer (pH 7.5). **b** Thermostability: 1.0 μg/mL purified enzyme was incubated at the specific temperature (50, 60, 70) for different times, and the residual activity was measured by standard assay. **c** pH optimum: the enzyme activity was assayed in various buffers (6.0–12.5) at 35 ℃. **d** pH stability: pH stability was measured by incubation of purified enzyme in different buffers (pH 6–10) for 24 h at 35 ℃, and the residual activity was measured by standard assay

The recombinant CbADH-6M was purified and the specific activity was detected to be 148 ± 1.5 U/mg at standard condition (35℃ and pH 7.5), which was slightly lower than the wild type. Then the activity of purified CbADH-6M was measured at various temperatures ranging from 15 to 75℃. As the temperature increased, the enzyme activity increased constantly, reaching the highest at 65℃ (Fig. 7a). Thermostability of the purified CbADH-6M was investigated at temperatures of 50, 60 and 70 ℃ (Fig. 7b), and the half-lives of the recombinant enzyme were measured to be 62.4 h, 4.9 h and 0.4 h (Additional file 1: Table S5), respectively. The optimum pH was determined by measuring the enzyme activities at different pH from 6.0–13.0 (Fig. 7c). The maximum activity was observed at pH of 9.5 (glycine–NaOH buffer). Investigation of pH stability showed that CbADH-6M remained relatively high activity from pH 6.0 to pH 10.0 (Fig. 7d).

### Regeneration of NADPH using recombinant CbADH-6M

**Fig. 8:**
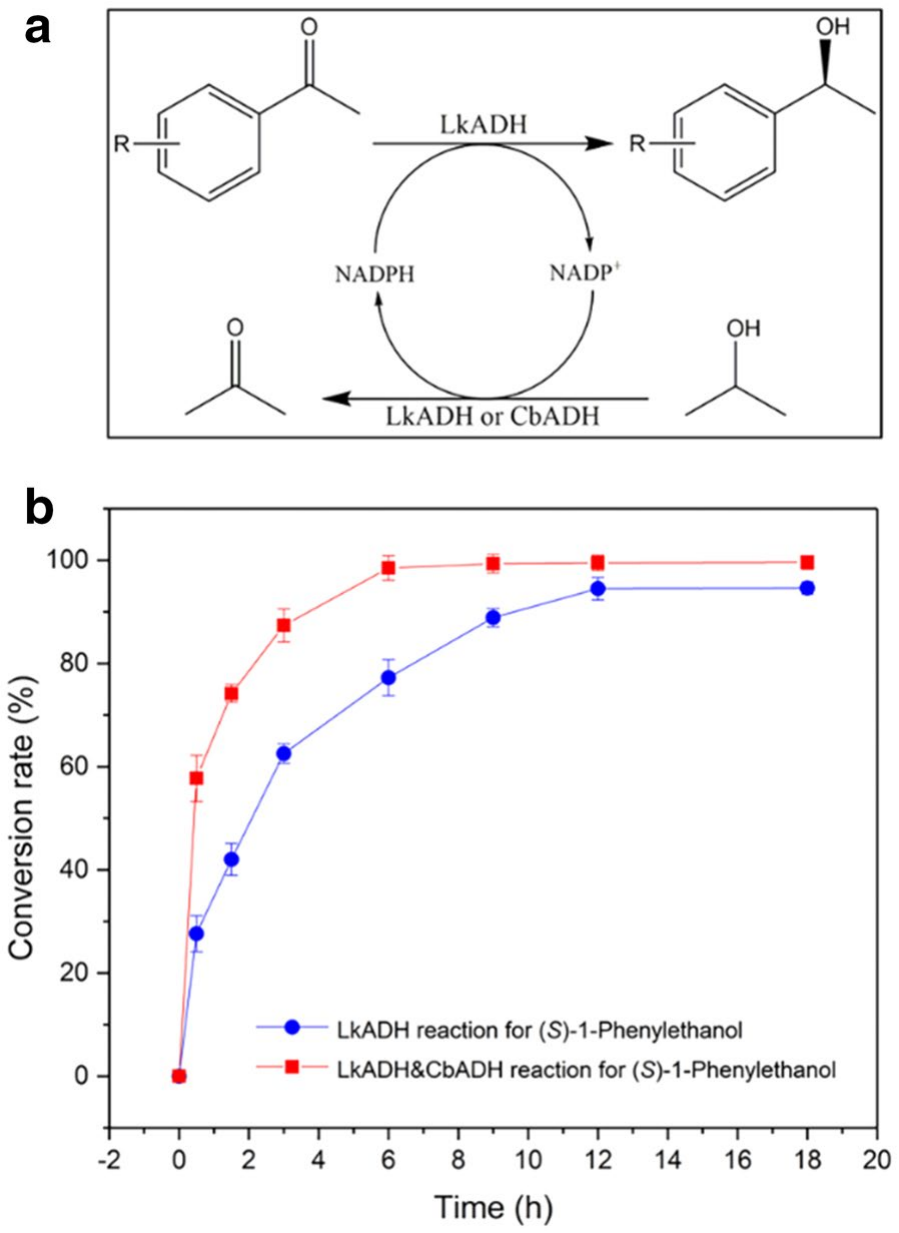
The reduction of acetophenone for synthesis of *(S)*-1-phenylethanol. **a** LkADH mono-enzymatic reaction. **b** LkADH and CbADH (CbADH-6M) dual enzyme catalyzed reaction

To explore the potential of CbADH at in situ NADPH regeneration, an engineered ADH from *Lactobacillus kefir* LkADH (He et al. 2015) coupled with CbADH-6M was employed in the synthesis of several fine chiral aromatic alcohols (Fig. 8a and Table 1). As shown in the reduction reaction for the synthesis of *(S)*-1-phenylethanol, if only LKADH was used, the reaction rate was low, and the conversion rate (Fig. 8b) hardly reached 100%. However, when the CbADH was added for the coenzyme recycle, the catalytic rate greatly accelerated. Additionally, in contrast to the reaction system catalyzed only by LkADH, the conversion rate of LkADH and CbADH reaction reached 100% within 6 h. Not only in the production of *(S)*-1-phenylethanol, but also in the synthesis of other important chiral aromatic alcohols (Table 1), reactions catalyzed by LkADH and CbADH exhibited much higher conversion rate than the LkADH mono-enzymatic reactions, indicating the great potential of CbADH-6M in industrial applications.

**Table 1:** Synthesis of chiral alcohols catalyzed by LkADH or LkADH and CbADH-6M

Entry	Substrate	Relative activity (%)	Product	ee (%)	Conversion rate (%)	
					LkADH	LkADH and CbADH-6M
1		100		(*S*) > 99	94.6	99.5
2		70		(*S*) > 99	80.3	99.5
3		92		(*S*) > 99	92.5	99.5
4		84		(*S*) > 99	86.1	99.5
5		59		(*S*) > 99	76.1	90.8

## Conclusions

In summary, CbADH with high stability and specific activity was identified for NADPH regeneration and a computational protein design named PROSS was applied for the soluble modification of this enzyme. The multipoint mutant CbADH-6M displayed a superb solubility and recombinant expression level than the wild type, with an enzyme activity of 46.3 U/mL, 16-fold improvement compared to the wild type in shake-flask fermentation and 2401.8 U/mL in two-phase high cell density fermentation. Additionally, the combining effect of multiple mutations in the variant improved the conformational stability of this protein, as well as the thermal stability. When coupling CbADH-6M (only 5% of total enzyme dosage) with LkADH, the catalytic rate of the reduction was greatly improved and the conversion rate was significantly higher than the reactions catalyzed by LkADH. The *ee* value of the product in the final reaction mixture was larger than 99%, showing a strict stereoselectivity. This CbADH-6M could be potentially used for the NADPH regeneration in the industrial synthesis of valuable compounds.

### Supplementary Information

**Additional file 1: Table S1.** Information of NADPH-dependent alcohol dehydrogenase. **Table S2**. Information of selected alcohol dehydrogenase. **Table S3.** The mutation sites of the PROSS mutants. **Table S4.** Thermal stability of the recombinant LkADH. **Table S5.** Activity half-life of the recombinant CbADH-6M. **Figure S1.** Multiple sequence alignment of alcohol dehydrogenases. **Figure S2.** SDS-PAGE analysis of the protein expression of the alcohol dehydrogenases. **Figure S3.** SDS-PAGE analysis of the protein expression at different inducing temperatures. **Figure S4**. SDS-PAGE analysis of the protein expression at different IPTG concentration. **Figure S5.** CbADH activity of molecular chaperones co-expression strains. **Fig S6**. SDS-PAGE analysis of protein expression of molecular chaperones co-expression strains. **Fig S7.** Multiple sequence alignment of CbADH mutant. **Fig S8.** SDS-PAGE analysis of protein expression of PROSS mutants. **Fig S9.** Protein purification of PROSS mutants. **Fig S10** HPLC spectrum of (S)-1-phenylethanol. **Fig S11.** HPLC spectrum of (S)-1-(4-methylphenyl)ethanol. **Fig S12.** HPLC spectrum of (S)-1-(4-fluorophenyl)ethanol. **Fig S13**. HPLC spectrum of (S)-1-(4-chlorophenyl)ethanol. **Fig S14**. HPLC spectrum of (S)-1-(3,4,5-trifluorophenyl)ethanol.

## Data Availability

The data and materials in this work are available from the corresponding author on reasonable request.

## Abbreviations

ADH: Alcohol dehydrogenase
CbADH: An alcohol dehydrogenase from *Clostridium beijerinckii*
LkADH: An alcohol dehydrogenase from *Lactobacillus kefir*
WT: Wild type
HPLC: High-performance liquid chromatography
