# Supplementary material for: Computational design of highly stable and soluble alcohol dehydrogenase for NADPH regeneration
Source: Bioresour Bioprocess. 2021 Feb 7;8(1):12. doi: 10.1186/s40643-021-00362-w (PMC10992930; doi:10.1186/s40643-021-00362-w)
Supplement: Supplementary file 1 — Additional file 1: Table S1. Information of NADPH-dependent alcohol dehydrogenase. Table S2. Information of selected alcohol dehydrogenase. Table S3. The mutation sites of the PROSS mutants. Table S4. Thermal stability of the recombinant LkADH. Table S5. Activity half-life of the recombinant CbADH-6M. Figure S1. Multiple sequence alignment of alcohol dehydrogenases. Figure S2. SDS-PAGE analysis of the protein expression of the alcohol dehydrogenases. Figure S3. SDS-PAGE analysis of the protein expression at different inducing temperatures. Figure S4. SDS-PAGE analysis of the protein expression at different IPTG concentration. Figure S5. CbADH activity of molecular chaperones co-expression strains. Fig S6. SDS-PAGE analysis of protein expression of molecular chaperones co-expression strains. Fig S7. Multiple sequence alignment of CbADH mutant. Fig S8. SDS-PAGE analysis of protein expression of PROSS mutants. Fig S9. Protein purification of PROSS mutants. Fig S10 HPLC spectrum of (S)-1-phenylethanol. Fig S11. HPLC spectrum of (S)-1-(4-methylphenyl)ethanol. Fig S12. HPLC spectrum of (S)-1-(4-fluorophenyl)ethanol. Fig S13. HPLC spectrum of (S)-1-(4-chlorophenyl)ethanol. Fig S14. HPLC spectrum of (S)-1-(3,4,5-trifluorophenyl)ethanol. [file 40643_2021_362_MOESM1_ESM.docx]

**Additional Information**

**Computational design of highly stable and soluble alcohol dehydrogenase for NADPH regeneration**

**Jinling Xu^1^, Haisheng Zhou^2^*****, Haoran Yu^1,2^, Tong Deng^1^, Ziyuan Wang^1^, Hongyu Zhang^1,2^, Jianping Wu^1,2^, Lirong Yang^1,2^***

*^1^ Institute of Bioengineering, College of Chemical and Biological Engineering, Zhejiang University, Hangzhou, 310027, China*

*^2^Hangzhou Global Scientific and Technological Innovation Center, Zhejiang University, Hangzhou, 310027, China*

contents

[Additional tables 2](#_Toc55066405)

[Table S1. Information of NADPH-dependent alcohol dehydrogenase. 2](#_Toc55066406)

[Table S2. Information of selected alcohol dehydrogenase. 3](#_Toc55066407)

[Table S3. The mutation sites of the PROSS mutants. 3](#_Toc55066408)

[Table S4. Thermal stability of the recombinant LkADH. 4](#_Toc55066409)

[Table S5. Activity half-life of the recombinant CbADH-6M. 4](#_Toc55066410)

[Additional figures 5](#_Toc55066411)

[Figure S1. Multiple sequence alignment of alcohol dehydrogenases. 5](#_Toc55066413)

[Figure S2. SDS-PAGE analysis of the protein expression of the alcohol dehydrogenases. 6](#_Toc55066414)

[Figure S3. SDS-PAGE analysis of the protein expression at different inducing temperatures. 7](#_Toc55066415)

[Figure S4. SDS-PAGE analysis of the protein expression at different IPTG concentration. 7](#_Toc55066416)

[Figure S5. CbADH activity of molecular chaperones co-expression strains. 8](#_Toc55066417)

[Fig S6. SDS-PAGE analysis of protein expression of molecular chaperones co-expression strains. 8](#_Toc55066418)

[Fig S7. Multiple Sequence alignment of CbADH mutant. 9](#_Toc55066419)

[Fig S8. SDS-PAGE analysis of protein expression of PROSS mutants. 9](#_Toc55066420)

[Fig S9. Protein purification of PROSS mutants. 9](#_Toc55066421)

[Fig S10 HPLC spectrum of (*S*)-1-phenylethanol. 10](#_Toc55066422)

[Fig S11. HPLC spectrum of (*S*)-1-(4-Methylphenyl)ethanol. 11](#_Toc55066423)

[Fig S12. HPLC spectrum of (*S*)-1-(4-Fluorophenyl)ethanol. 12](#_Toc55066424)

[Fig S13. HPLC spectrum of (*S*)-1-(4-Chlorophenyl)ethanol. 13](#_Toc55066425)

[Fig S14. HPLC spectrum of (*S*)-1-(3,4,5-trifluorophenyl)ethanol. 14](#_Toc55066426)

[References 15](#_Toc55066427)

# Additional tables

## Table S1. Information of NADPH-dependent alcohol dehydrogenase.

| alcohol dehydrogenase | Source | Specific activity (U/mg protein) | Enzyme assay temperature | Reference |  |
| --- | --- | --- | --- | --- | --- |
| TgADH | *Thermococcus guaymasensis* | 1011^a^ | 80℃ | ^(Ying & Ma, 2011)^ |  |
| MtADH | *Methanogenium thermophilum* | 176^a^ | 40℃ | ^(Widdel & Wolfe, 1989)^ |  |
| CbADH | *Clostridium beijerinckii* | 140^a^ | 25℃ | ^(Ismaiel et al., 1993)^ | |
| TbADH | *Thermoanaerobium brockii* | 63^a^ | 40℃ | ^(Peretz et al., 1997)^ |  |
| TeADH | *Thermoanaerobacter ethanolicus* | 40^a^ | 60℃ | ^(Burdette & Zeikus, 1994)^ |  |
| EhADH | *Entamoeba histoytica* | 29 ± 10^a^ | 25℃ | ^(Kumar et al., 1992)^ |  |
| LkADH | *Lactobacillus kefir* | 7.58^a^ | 20℃ | ^(He et al., 2015)^ |  |
| ThADH | *Thermotoga hypogea* | 298 ± 6^b^ | 80℃ | ^(Ying et al., 2007)^ |  |
| AsADH | *Acinetobacter* sp. | 224^b^ | 30℃ | ^(Akio Tani, 2000)^ |  |
| TsADH | *Thermococcus* strain ES1 | 120.6 ± 3.2^b^ | 80℃ | ^(Ying et al., 2009)^ |  |
| PfADH | *Pyrococcus furiosus* | 28.5^b^ | 80℃ | ^(Ma & Adams, 1999)^ |  |
| ScADH | *Saccharomyces cerevisiae* | 12.8^b^ | 25℃ | ^(Carol Larroy, 2002)^ |  |

a: specific activity detected with 2-propanol;

b: specific activity detected with 1-propanol.

## Table S2. Information of selected secondary alcohol dehydrogenase.

| alcohol dehydrogenase | Source | NCBI accession number | Theoretical molecular weight (kDa) |
| --- | --- | --- | --- |
| TgADH | *Thermococcus guaymasensis* | WP_062374090.1 | 39.2 |
| MtADH | *Methanogenium thermophilum* | CAA77275.1 | 37.2 |
| CbADH | *Clostridium beijerinckii* | WP_077844196.1 | 37.7 |
| TbADH | *Thermoanaerobium brockii* | CAA46053.1 | 37.6 |
| TeADH | *Thermoanaerobacter ethanolicus* | ABC50090.1 | 37.6 |
| EhADH | *Entamoeba histoytica* | BAN37960.1 | 39.2 |
| LkADH | *Lactobacillus kefir* | AAP94029.1 | 26.8 |

## Table S3. The mutation sites of the PROSS mutants.

| mutant | mutation sites |
| --- | --- |
| CbADH-WT | - |
| CbADH-6M | S24P，G182A，G196A，H222D，S250E，S254R |
| CbADH-10M | S24P，G182A，G196A，H222D，S250E，S254R，E18P，G121A，L335R，A347V |
| CbADH-14M | S24P，G182A，G196A，H222D，S250E，S254R，E18P，G121A，L335R，A347V，K138D，N144Q，V310Q，Y324F |
| CbADH-19M | S24P，G182A，G196A，H222D，S250E，S254R，E18P，G121A，L335R，A347V，K138D，N144Q，V310Q，Y324F，L32T，K53H，I135H，N312G，V323R |
| CbADH-24M | S24P，G182A，G196A，H222D，S250E，S254R，E18P，G121A，L335R，A347V，K138D，N144Q，V310Q，Y324F，L32T，K53H，I135H，N312G，V323R，N10G，F45W，D139G，A302M，L351I |

## Table S4. Thermal stability of the recombinant LkADH.

| Temperature (°C) | Half-life of activity (h) |
| --- | --- |
| 20 | 55.4 |
| 30 | 26.9 |
| 40 | 5.8 |
| 50 | 1.0 |

## Table S5 Activity half-life of the recombinant CbADH-6M.

| Temperature (°C) | Half-life of activity (h) |
| --- | --- |
| 50 | 62.4 |
| 60 | 4.9 |
| 70 | 0.4 |

# Additional figures


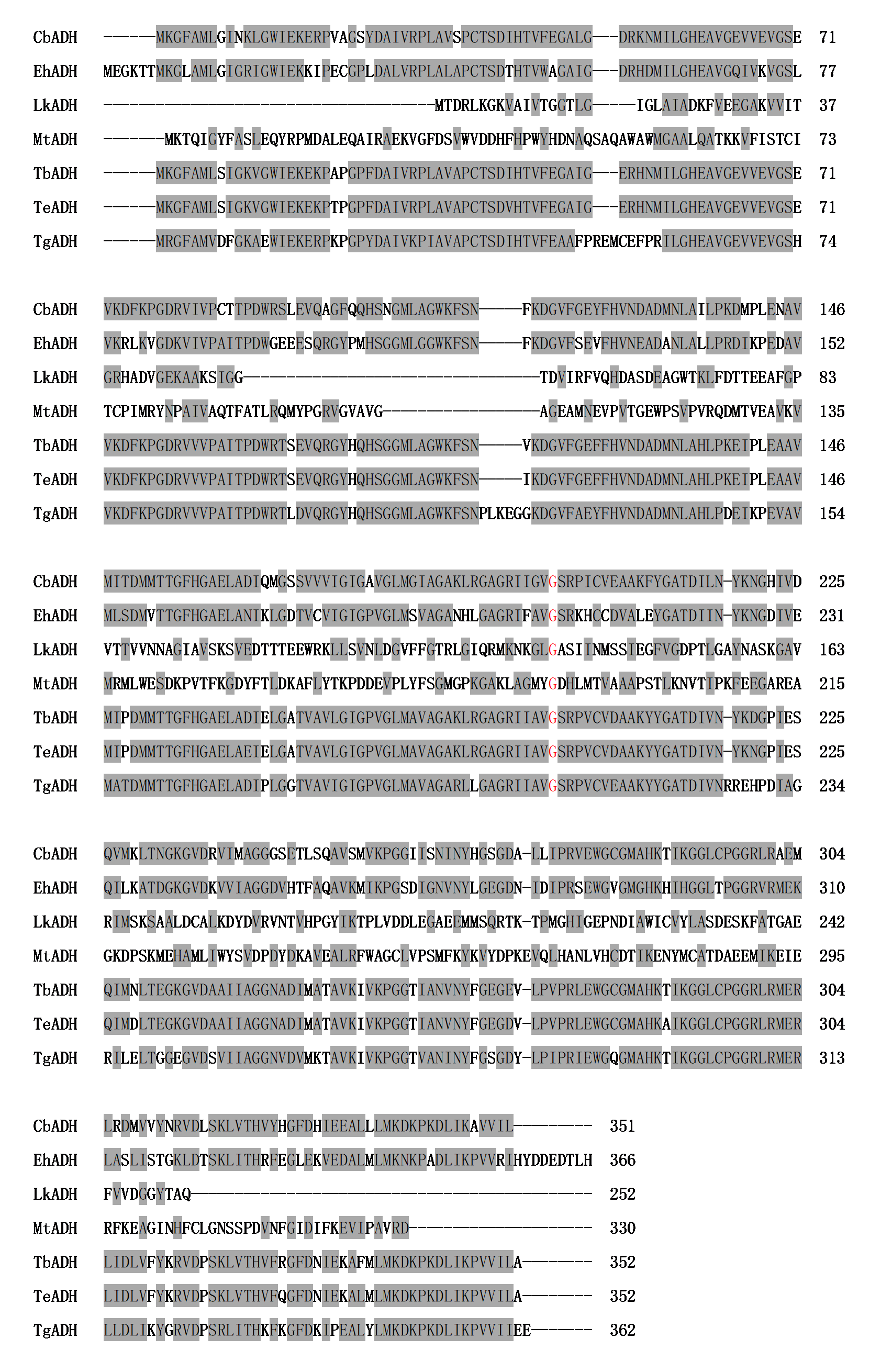


## Figure S1. Multiple sequence alignment of alcohol dehydrogenases.


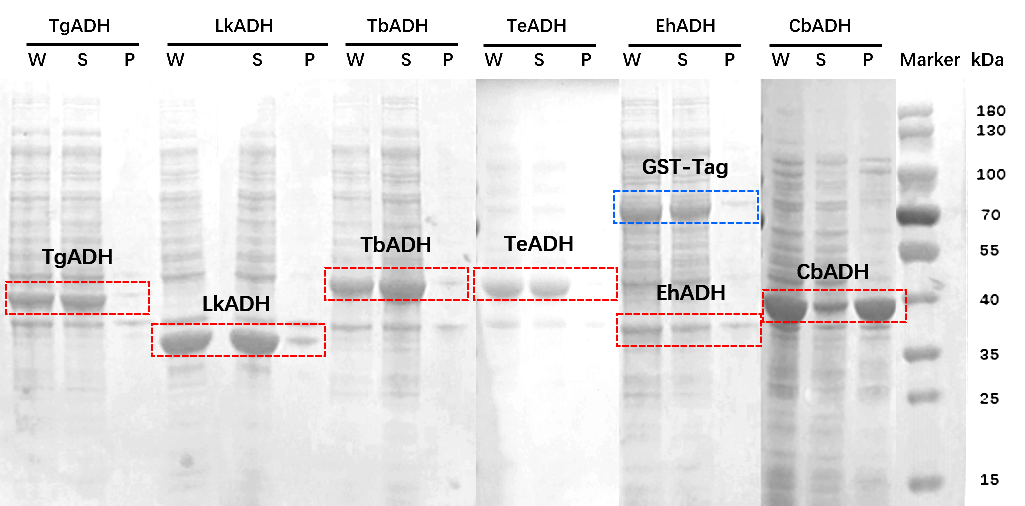


## Figure S2. SDS-PAGE analysis of the protein expression of the alcohol dehydrogenases. Lane M: molecular weight marker; Lane W: whole cell protein; Lane S: supernatant; Lane P: precipitation.


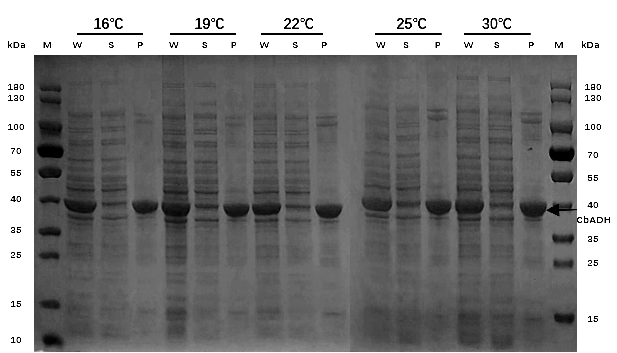


## Figure S3. SDS-PAGE analysis of the protein expression of CbADH (wild type) at different inducing temperatures. The expected size of CbADH is indicated by black arrow.


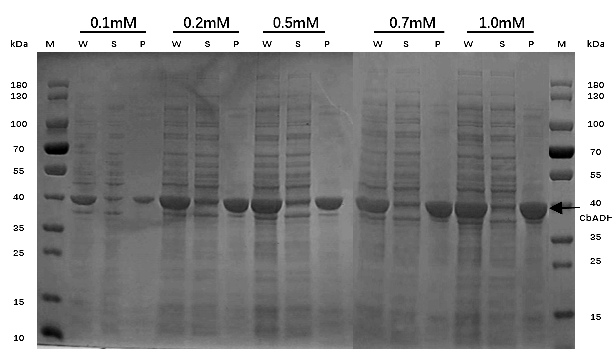


## Figure S4. SDS-PAGE analysis of the protein expression of CbADH (wild type) at different IPTG concentration and 25°C.


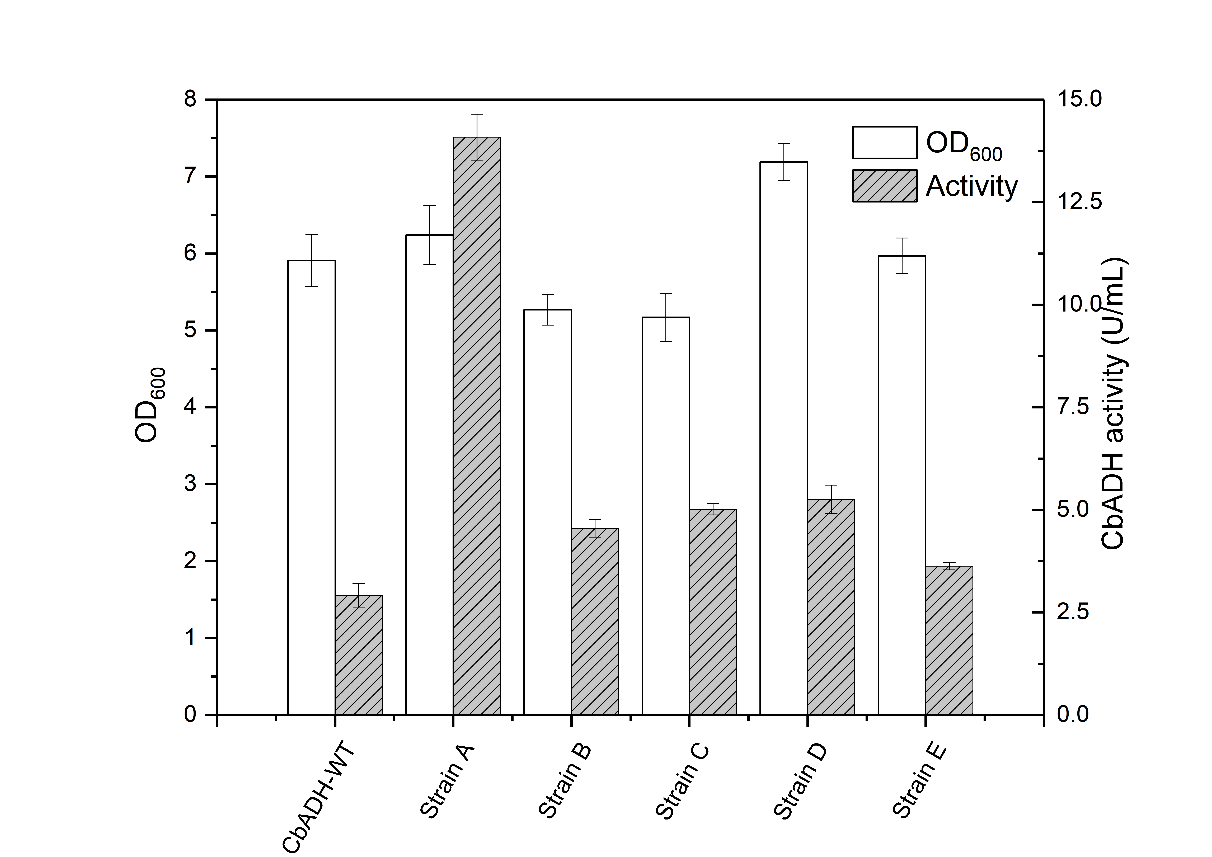


## Figure S5. CbADH activity of molecular chaperones co-expression strains. Molecular chaperones co-expressed in Strains: Strain A: pGro7/GroES-GroEL; Strain B: pKJE7/DnaK-DnaJ-GrpE; Strain C: pG-KJE7/DnaK-DnaJ-GrpE- GroES-GroEL; Strain D: pG-Tf2/GroES-GroEL-Tf; Strain E: pTf16/Tf. (Plasmid/molecular chaperones).


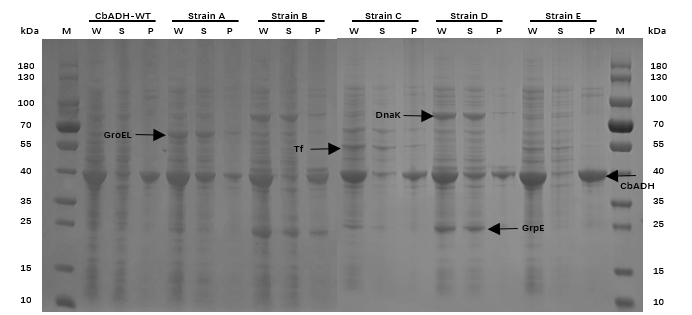


## Fig S6. SDS-PAGE analysis of protein expression of molecular chaperones co-expression strains. Molecular chaperones co-expressed in Strains: Strain A: pGro7/GroES-GroEL; Strain B: pKJE7/DnaK-DnaJ-GrpE; Strain C: pG-KJE7/DnaK-DnaJ-GrpE- GroES-GroEL; Strain D: pG-Tf2/GroES-GroEL-Tf; Strain E: pTf16/Tf. (Plasmid/molecular chaperones). The expected size of molecular chaperones are indicated by black arrow. GroEL(60 kDa), Tf (56 kDa), DnaK (70 kDa), GrpE (22 kDa).


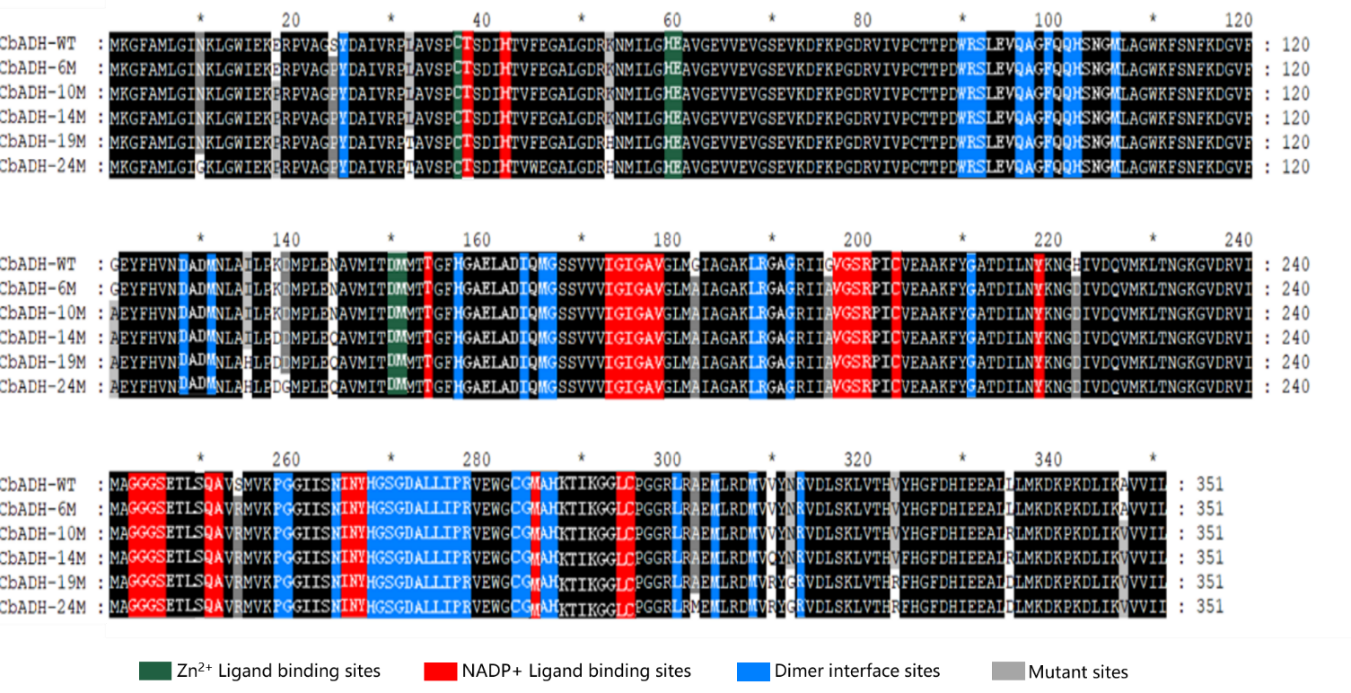


## Fig S7. Multiple Sequence alignment of the CbADH wild type and mutants.


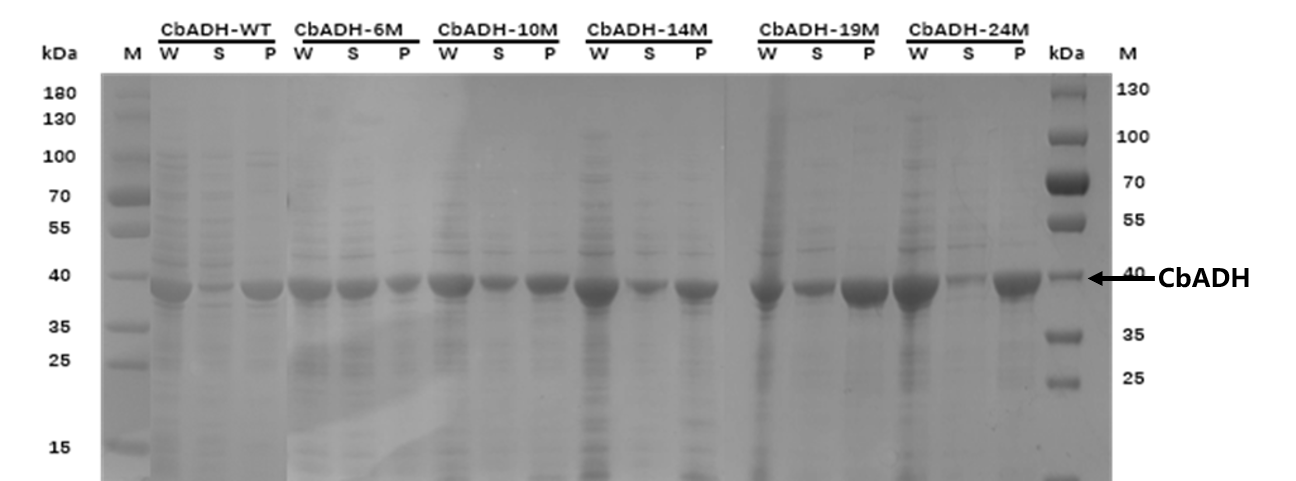


## Fig S8. SDS-PAGE analysis of protein expression of the CbADH wild type and mutants.


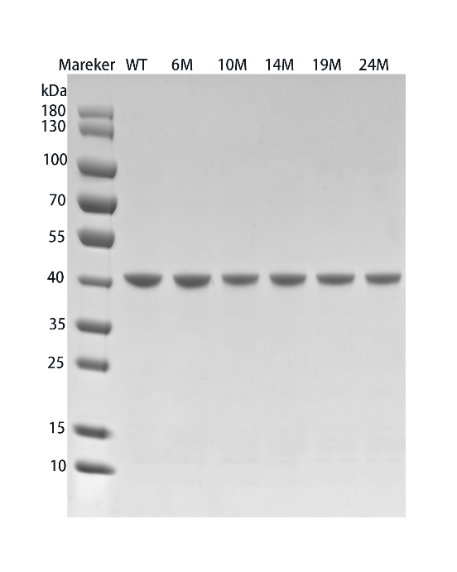


## Fig S9. Protein purification of the CbADH wild type and mutants.


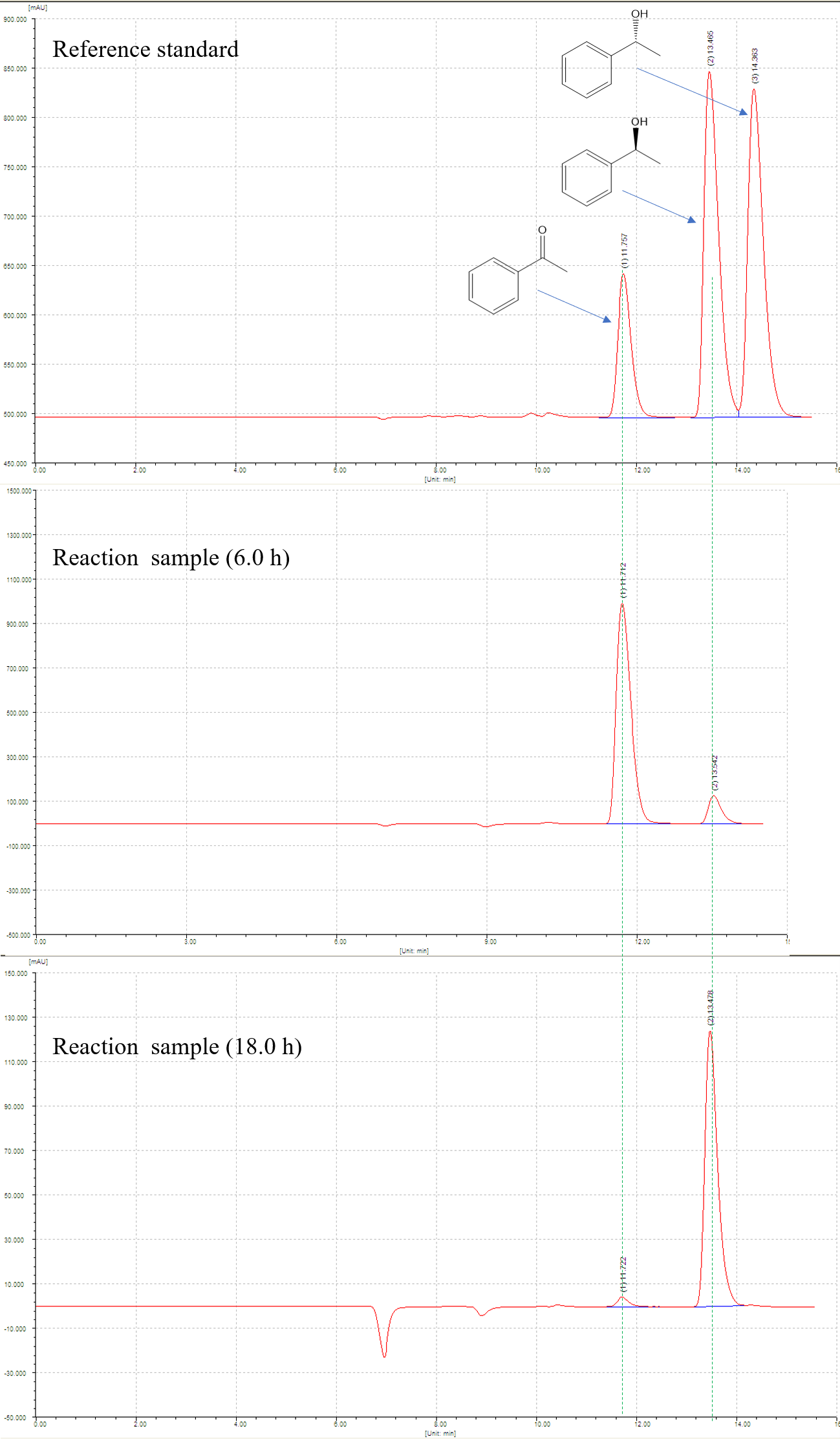


## Fig S10 HPLC spectrum of *(S)*-1-phenylethanol synthesis reaction.


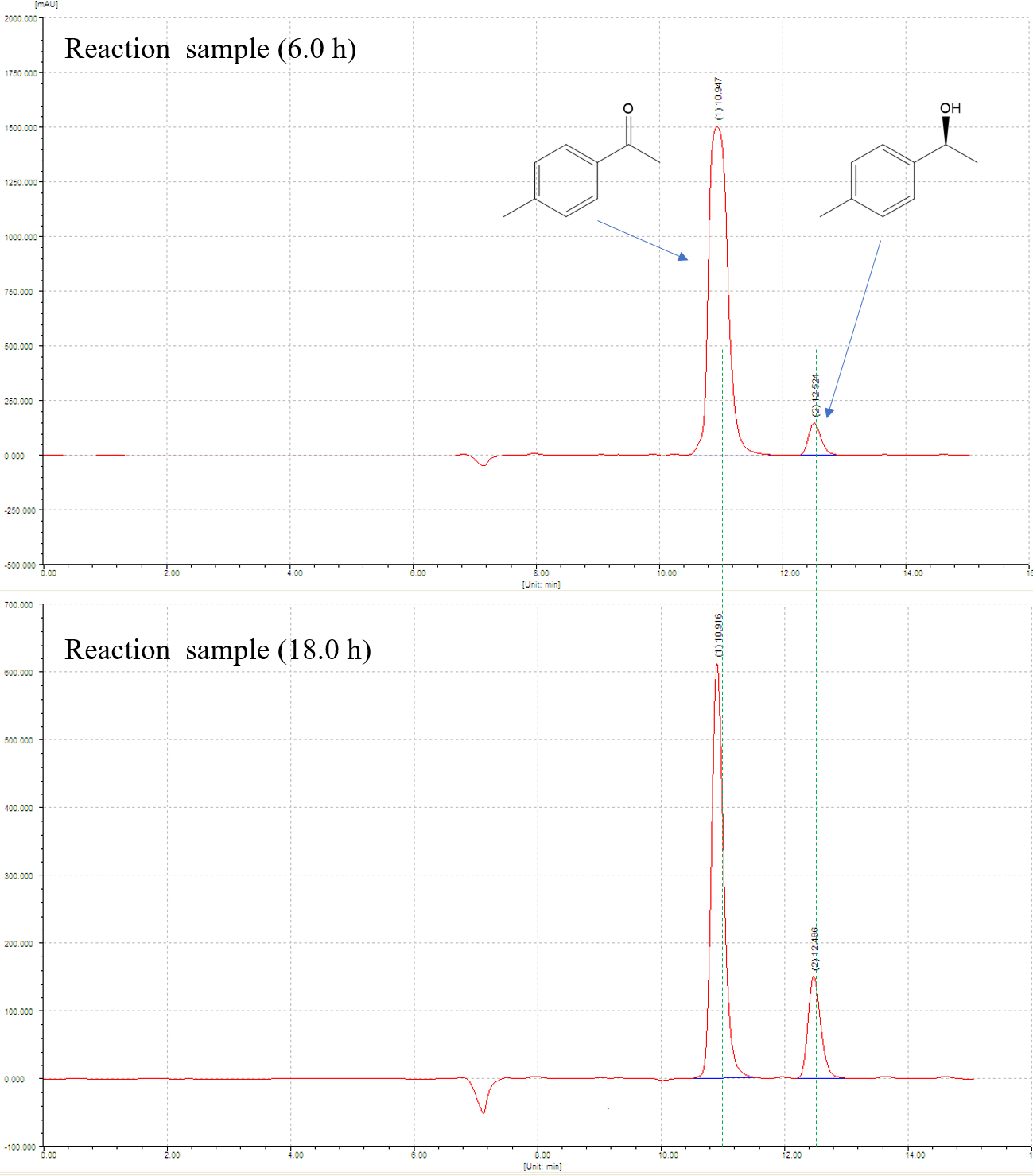


## Fig S11. HPLC spectrum of (*S*)-1-(4-Methylphenyl)ethanol synthesis reaction.


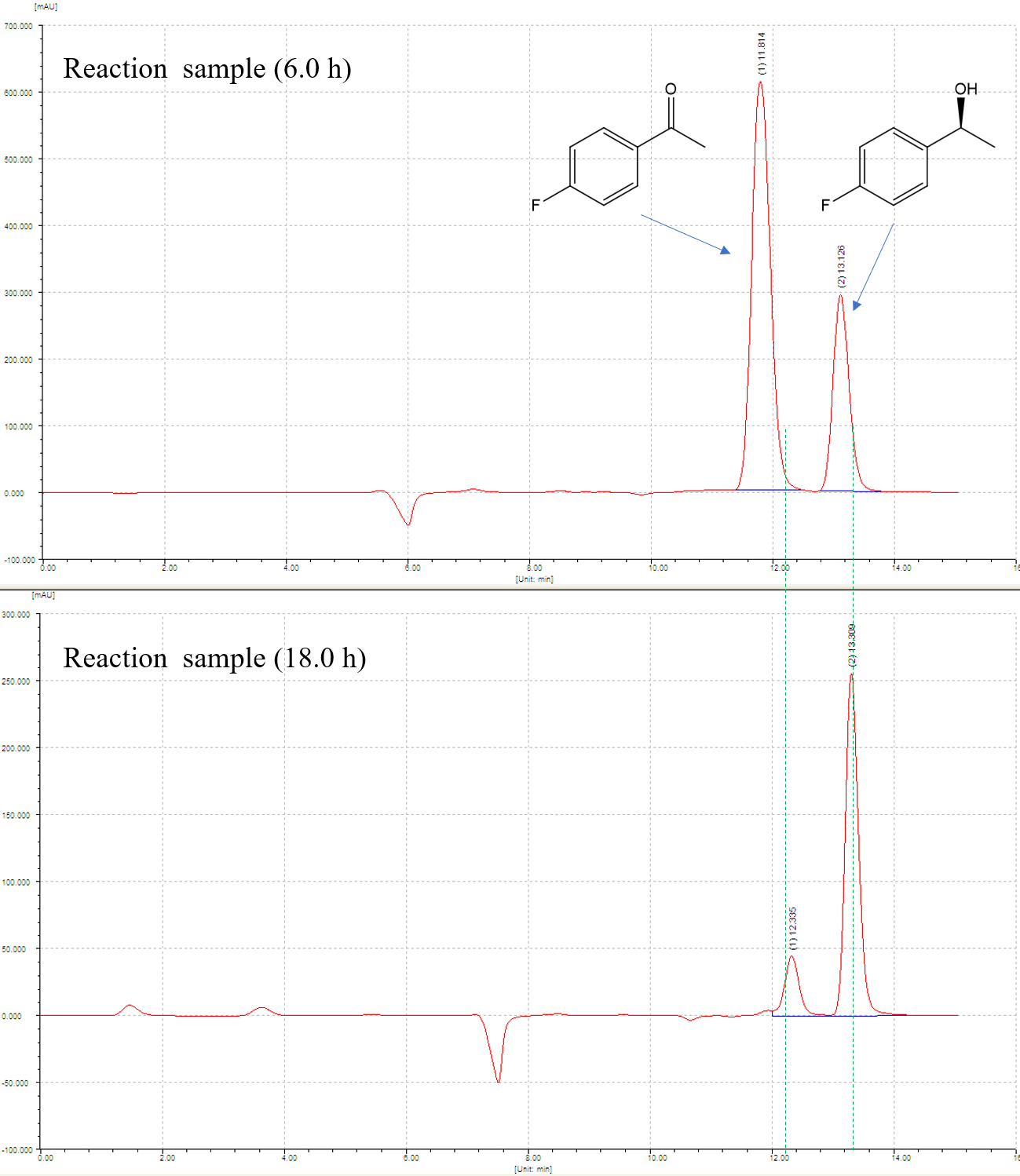


## Fig S12. HPLC spectrum of (*S*)-1-(4-Fluorophenyl)ethanol synthesis reaction.


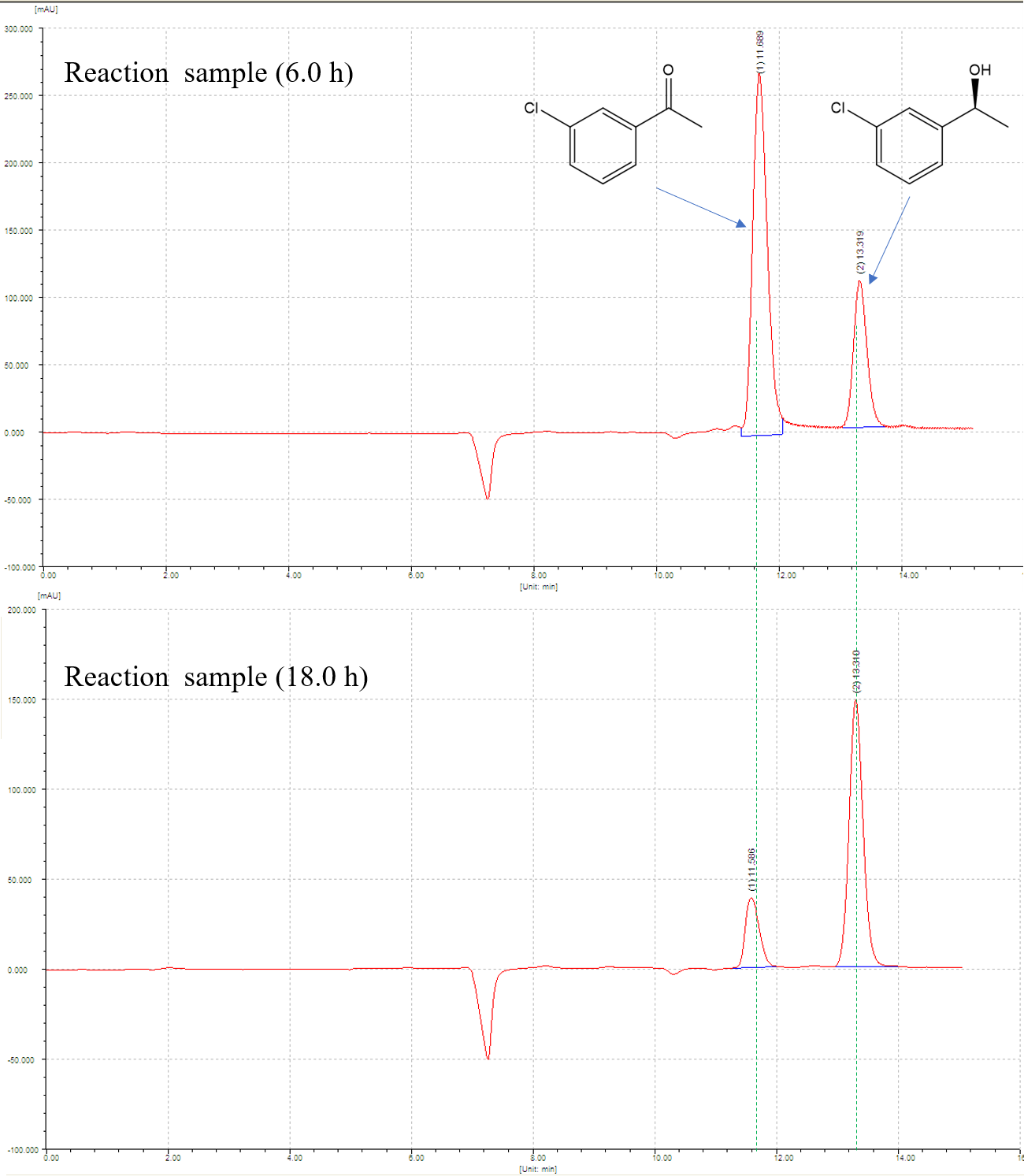


## Fig S13. HPLC spectrum of (*S*)-1-(4-Chlorophenyl)ethanol synthesis reaction.


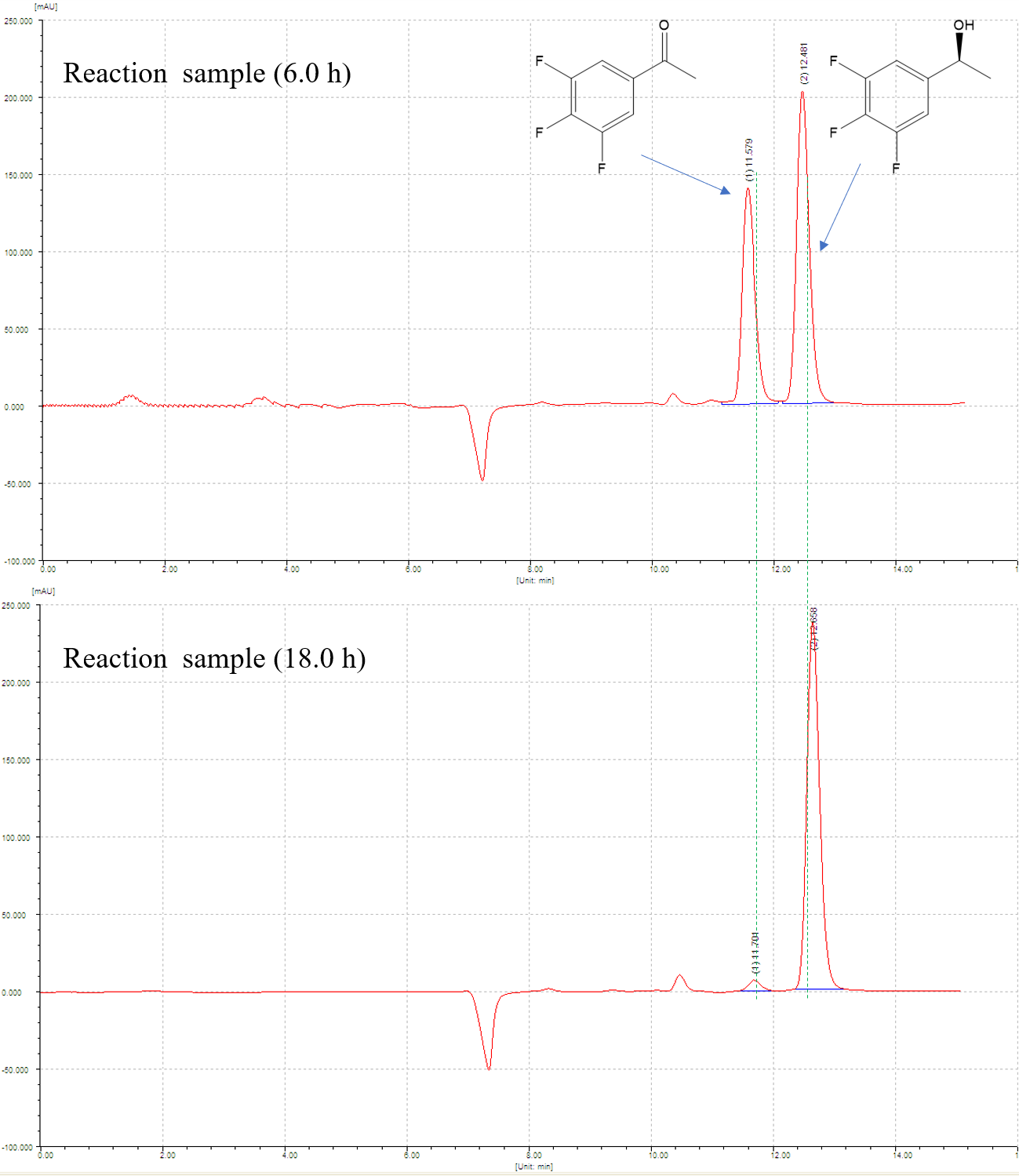


## Fig S14. HPLC spectrum of (*S*)-1-(3,4,5-trifluorophenyl)ethanol synthesis reaction.

# References

Akio Tani, Y.S., Takeru Ishige, Nobuo Kato. 2000. Thermostable NADP(+)-dependent medium-chain alcohol dehydrogenase from *Acinetobacter* sp. strain M-1 purification and characterization and gene expression in *Escherichia coli*. *Applied and Environmental Microbiology*, **66**(12), 5231-5235.

Burdette, D., Zeikus, J.G. 1994. Purification of acetaldehyde dehydrogenase and alcohol dehydrogenases from Thermoanaerobacter ethanolicus 39E and characterization of the secondary-alcohol dehydrogenase (2° Adh) as a bifunctional alcohol dehydrogenase-acetyl-CoA reductive thioesterase. *Biochemical Journal*, **302**(1), 163-170.

Carol Larroy, M.R.F., Eva González, Xavier Parés, and Josep A Biosca. 2002. Characterization of the *Saccharomyces cerevisiae* YMR318C (ADH6) gene product as a broad specificity NADPH-dependent alcohol dehydrogenase: relevance in aldehyde reduction. *Biochemical journal*, **361**, 163–172.

He, X.J., Chen, S.Y., Wu, J.P., Yang, L.R., Xu, G. 2015. Highly efficient enzymatic synthesis of tert-butyl (S)-6-chloro-5-hydroxy-3-oxohexanoate with a mutant alcohol dehydrogenase of Lactobacillus kefir. *Appl Microbiol Biotechnol*, **99**(21), 8963-75.

Ismaiel, A.A., Zhu, C.X., Colby, G.D., Chen, J.S. 1993. Purification and characterization of a primary-secondary alcohol dehydrogenase from two strains of Clostridium beijerinckii. *Journal of Bacteriology*, **175**(16), 5097-5105.

Kumar, A., Shen, P.S., Descoteaux, S., Pohl, J., Bailey, G., Samuelson, J. 1992. Cloning and expression of an NADP(+)-dependent alcohol dehydrogenase gene of Entamoeba histolytica. *Proceedings of the National Academy of Sciences*, **89**(21), 10188.

Ma, K., Adams, M.W. 1999. An unusual oxygen-sensitive, iron- and zinc-containing alcohol dehydrogenase from the hyperthermophilic archaeon Pyrococcus furiosus. *Journal of bacteriology*, **181**(4), 1163-1170.

Peretz, M., Bogin, O., Tel-Or, S., Cohen, A., Li, G., Chen, J.-S., Burstein, Y. 1997. Molecular Cloning, Nucleotide Sequencing, and Expression of Genes Encoding Alcohol Dehydrogenases From the ThermophileThermoanaerobacter brockiiand the MesophileClostridium beijerinckii. *Anaerobe*, **3**(4), 259-270.

Widdel, F., Wolfe, R.S. 1989. Expression of secondary alcohol dehydrogenase in methanogenic bacteria and purification of the F420-specific enzyme from Methanogenium thermophilum strain TCI. *Archives of Microbiology*, **152**(4), 322-328.

Ying, X., Grunden, A.M., Nie, L., Adams, M.W., Ma, K. 2009. Molecular characterization of the recombinant iron-containing alcohol dehydrogenase from the hyperthermophilic Archaeon, Thermococcus strain ES1. *Extremophiles*, **13**(2), 299-311.

Ying, X., Ma, K. 2011. Characterization of a zinc-containing alcohol dehydrogenase with stereoselectivity from the hyperthermophilic archaeon Thermococcus guaymasensis. *Journal of bacteriology*, **193**(12), 3009-3019.

Ying, X., Wang, Y., Badiei, H.R., Karanassios, V., Ma, K. 2007. Purification and characterization of an iron-containing alcohol dehydrogenase in extremely thermophilic bacterium Thermotoga hypogea. *Arch Microbiol*, **187**(6), 499-510.
